# Supplementary material for: Effect of SARS-CoV-2 Vaccination on Symptoms from Post-Acute Sequelae of COVID-19: Results from the Nationwide VAXILONG Study
Source: Vaccines (Basel). 2021 Dec 30;10(1):46. doi: 10.3390/vaccines10010046 (PMC8781023; doi:10.3390/vaccines10010046)
Supplement: Supplementary file 1 [file vaccines-10-00046-s001.zip › vaccines-1505954-supplementary.pdf]

## Appendix: Questionnaire

**Évolution à long terme du COVID-long et effets potentiels de la vaccination sur les symptômes**

Bonjour,

Ce questionnaire anonyme réalisé par l'équipe de rhumatologie du CHU de Strasbourg s'inscrit dans la suite d'un travail de recherche sur des patients atteints de COVID-long suivis à Strasbourg (publication disponible en accès libre https://link.springer.com/article/10.1007/s40121-021-00484-w).

Dans l'objectif de mieux comprendre l'évolution à long terme des symptômes de COVID-long et des effets potentiels de la vaccination contre le COVID-19 sur les symptômes, nous vous proposons de remplir ce sondage anonyme. Vous pouvez le remplir même si vous n'avez pas été vacciné(e) contre le COVID-19.

Il est nécessaire de prévoir environ 5 minutes pour le remplir complètement. En effet, sans informations précises, l'interprétation des résultats ne serait pas possible et ne pourrait pas profiter à l'ensemble de la Communauté Scientifique et COVID-long.

Merci à vous,
L'équipe de rhumatologie du CHU de Strasbourg

Les informations collectées dans ce questionnaire sont anonymes et enregistrées dans une base de données informatique par Marc SCHERLINGER et Renaud FELTEN pour le Service de Rhumatologie de Strasbourg et le Centre National de Référence des maladies auto-immunes et systémiques rares (RESO). Les données collectées seront traitées sur la base légale du consentement.

Les données recueillies seront communiquées uniquement aux destinataires suivants : Marc SCHERLINGER et Renaud FELTEN, du Service de Rhumatologie de Strasbourg, et Centre National de Référence des maladies auto-immunes et systémiques rares (RESO). Elles seront conservées pendant la durée de l'étude.

Vous pouvez accéder à vos données, les rectifier, exiger leur suppression, ou exercer votre droit de limiter l'utilisation de vos données. Vous pouvez retirer votre consentement à l'analyse de vos données à tout moment.

Vous pouvez consulter le site du cnil.fr pour plus d'informations sur vos droits.

Pour exercer vos droits, ou si vous avez des questions sur l'analyse de vos données dans cette étude, vous pouvez contacter :
Dr Marc SCHERLINGER
Service de Rhumatologie de Strasbourg
1 avenue Molière
67098 STRASBOURG Cedex

## CONSENTEMENT

Acceptez-vous que les réponses de ce sondage soient utilisées à des fins de recherche médicale ?
Oui
Non

## DEMOGRAPHIE

Quel âge avez-vous ?

Quel est votre sexe ?

Quelle est votre situation professionnelle ? (liste déroulante)

## VOTRE INFECTION INITIALE

De quand date votre COVID-19 initiale ? (Si vous ne connaissez pas le jour exact, indiquez la date approximative)

Quels symptômes avez-vous présenté initialement? (liste déroulante)

Comment a été gérée votre infection initiale ? (liste déroulante)

Avez-vous présenté une perte d'odorat ou de goût (anosmie/agueusie) prolongée de survenue brutale ?
Oui
Non

Avez-vous bénéficié d'un test RT-PCR à la recherche du virus SARS-CoV-2 dans les 2 premières semaines suivant le début des symptômes ?

Avez-vous bénéficié d'un test antigénique à la recherche du virus SARS-CoV-2 dans les 2 premières semaines suivant le début des symptômes ?

Avez-vous bénéficié d'un scanner pulmonaire après le début de vos symptômes ?

Avez-vous bénéficié d'une sérologie SARS-CoV-2 dans les suites de votre infection initiale (indiquez alors le résultat de la première sérologie) ?

## EVOLUTION DE VOTRE SEROLOGIE

Votre sérologie COVID-19 s'est-elle ensuite négativée (avant potentielle vaccination) ?

## VOTRE COVID-LONG

Souffrez-vous de symptômes prolongés au-delà de 4 semaines suivant le début de la phase aiguë de la COVID-19 ?
Oui
Non

Vos symptômes initiaux et prolongés sont-ils NON expliqués par un autre diagnostic sans lien connu avec la COVID-19 ?

Y-a-t-il eu un intervalle libre entre votre infection initiale et l'apparition des symptômes prolongés ? Si oui, indiquer l'intervalle en jours dans la colonne "autre" ("X" jours).
Oui
Non

Vos symptômes sont-ils fluctuants en intensité ou en diversité ?
Oui
Non

Quels symptômes de COVID-long avez-vous identifié ? (liste déroulante)

En moyenne, sur les dernières semaines vos symptômes prolongés...
Ont tendance à s'améliorer
Ont tendance à s'aggraver
Ont identiques au début du COVID-long

## VACCINATION

Quel vaccin avez-vous reçu ? (liste déroulante)

Aviez-vous encore des symptômes de COVID-long lorsque vous avez été vacciné ?

Combien de doses de vaccin avez-vous reçu ?

Quand avez-vous reçu votre première dose ?

Quand avez-vous reçu votre deuxième dose (si vous n'en avez pas eu, veuillez ne pas répondre).

Quelle(s) étai(en)t votre (vos) raison(s) principale(s) de vous faire vacciner contre la COVID-19 ? (liste déroulante)

## EFFET DE LA VACCINATION SUR VOTRE COVID-LONG ET TOLERANCE

La vaccination a-t-elle eu un effet sur vos symptômes de COVID-long ?

Pour chacun de ces symptômes de COVID-long, veuillez indiquer les éventuelles modifications dans les suites de votre vaccination. (liste déroulante)

Quelle a été la durée de l'amélioration (ou de l'aggravation) suite à la vaccination ?

A quel moment est survenue l'amélioration (ou l'aggravation) ?

Avez-vous bénéficié d'une sérologie APRES votre dernière dose de vaccin ?

Commentaire libre sur l'effet de la vaccination sur vos symptômes de COVID-long

**NON VACCINATION**

Parmi ces propositions, quelle est celle qui exprime le mieux le fait que vous ne soyez pas vacciné ?
COVID-19 contractée il y a peu de temps
Peur des effets indésirables du vaccin
Peur d'aggraver mes symptômes de COVID-long
J'estime que ce n'est pas nécessaire
Le vaccin est contre-indiqué à cause du COVID-long
Pas assez de recul sur les vaccins
Autre à préciser

**AUTRE TRAITEMENT**

Avez-vous testé des traitements pour le COVID-long ? Si oui, veuillez les indiquer ainsi que leur efficacité dans votre cas ?

**MERCI DE VOTRE AIDE DANS CE TRAVAIL.**
Commentaire libre sur votre vécu du COVID-long (optionnel).
